# 

**Published:** 1892-06

**Authors:** 


### CONTENTS.

### COMMUNICATIONS.

Synopsis of a Lecture on Syphilis before the Classes in the Dental College of the University of Michigan........ 255
Blackening of the Teeth by Antipyrine........ 262
Valedictory Address, Tenth Annual Commencement (Dental Dept.) State University of Iowa........ 263
Tumors of the Mouth........ 270
Management of Practice........ 274
Various Kinds of Dentition........ 278
Contentment........ 279
An Overdose of Gold........ 283

### PROCEEDINGS.

Seventh District Dental Society of Ohio........ 284
Northern Ohio Dental Asssociation........ 286

### SELECTIONS.

On the Teaching of Anatomy to Advanced Medical Students........ 287
Pain and Inflammation of Dental Origin........ 294
Brain Rest and Sleep........ 296
The Ethics of Gum-Chewing........ 297
Human and Animal Blood........ 298
The Ancestry of Chalicotherium........ 299
How Medicines Act........ 299
Artificial Production of Variations in Types........ 300
An M.D. Self-Qualified........ 300
Nausea following Anæsthesia........ 301

### CORRESPONDENCE.

West Va. State Dental Society........ 301
Kentucky State Dental Association........ 302

### EDITORIAL.

The World's Columbian Exposition........ 303
Bibliographical........ 305
Hymeneal........ 306

